# The Silkworm Coming of Age—Early

**DOI:** 10.1371/journal.pgen.1002591

**Published:** 2012-03-08

**Authors:** René Feyereisen, Marek Jindra

**Affiliations:** 1 INRA-CNRS-Université de Nice Sophia Antipolis, Sophia Antipolis, France; 2 Biology Center, Academy of Sciences of the Czech Republic, Ceske Budejovice, Czech Republic; Janelia Farm Research Campus, Howard Hughes Medical Institute, United States of America

In order to grow, immature insects must periodically synthesize a new cuticle and shed the old one (a process of molting and ecdysis) until they have reached a stage permitting metamorphosis to the reproductive adult. Each molt is induced by a pulse of ecdysteroids, but the nature of the molt is determined by the juvenile hormones (JHs) [1]. These are sesquiterpenoids, synthesized from three isoprene units via the mevalonate pathway and decorated by an epoxide group on one end and a methyl ester on the other [1]. The JHs maintain larval characters, a status quo effect that is lifted when the level of JH drops. A threshold size must be attained for each molt and for metamorphosis to occur, and this size may relate to the limit of oxygen supply by the tracheal system [2], [3]. The number of molts varies among and sometimes within species; it is influenced by nutrition and by environmental and genetic signals. Yet, the mechanisms that “measure size” or “count the larval instars” are overridden by the experimental depletion of JHs. It has long been a tenet of insect endocrinology that removal of the corpora allata (CA), the endocrine glands that produce the JHs, causes precocious metamorphosis [1]. However, bringing the JH titer down experimentally is not as trivial as it may seem.

**Figure 1:**
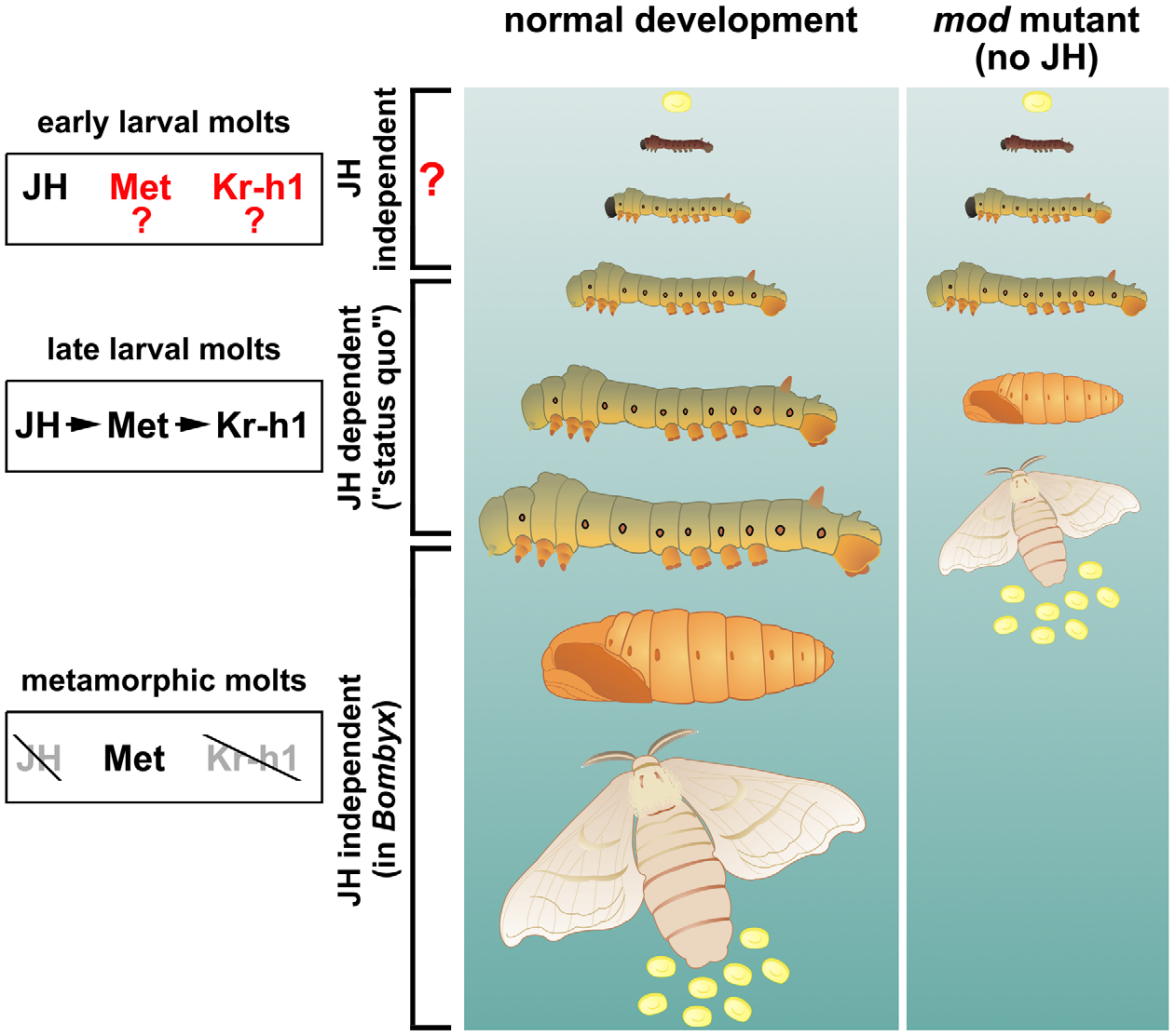
Development of the silkworm without juvenile hormones (JHs). Maintenance of larval characters before the larval-pupal and pupal-adult molts is the classical status quo role of JH. This JH effect is carried out through its receptor Met and the transcription factor Kr-h1. The *mod* mutants studied by Daimon et al. [4] lack a P450 enzyme (epoxidase) and cannot make functional JH, yet they do not enter metamorphosis until after two larval molts, producing precocious pupae instead of the normal fourth larval instar. This unexpected result suggests that the early larval instars might be insensitive to the lack of JH. Illustration: Martina Hajduskova.

In this issue, Daimon et al. [4] utilize the genetic resources of the silkworm *Bombyx mori* to explore why larvae of the *dimolting (mod)* mutant strain undergo metamorphosis early, giving miniature pupae and adults after just three instars rather than the normal five (Figure 1). They identified this *mod* trait as a gene for a cytochrome P450 enzyme, CYP15C1, the silkworm ortholog of a previously identified JH epoxidase of the cockroach CA [5]. Daimon et al. show that the *mod* mutant is a null caused by a deletion that truncates CYP15C1. The mutation can be rescued by transgenic production of the wild type enzyme in the CA using the GAL4-UAS system, or by topical application of a JH agonist. Biochemical experiments confirm that CYP15C1 is a stereospecific epoxidase expressed specifically in the CA throughout postembryonic development.

The work of Daimon et al. is forceful in its elegance, and it brings a surprise: the CA of *mod* mutants do not produce the JHs normally needed for the status quo of larval development, yet the *mod* larvae do not commit to become pupae until the third larval instar (Figure 1). Might this work break the status quo of insect endocrinology? Daimon et al. propose that authentic (epoxidized) JHs are essential for the classic status quo but not in early larval stages (1 and 2) in *Bombyx*. It turns out that previous work corroborates this statement, and that the morphogenetic function of JHs in early larval instars of insects now requires closer attention.

There are different ways to remove JHs. Removal of the CA (“allatectomy”) is possible in some insects when there is a conjunction of skilled microsurgery with favorable size and anatomy of the glands. Thus, the steady hands and patience of Bounhiol [6] and Fukuda [7] succeeded in obtaining miniature pupae of the silkworm after operating on third-instar larvae, but they could not operate on earlier instars with smaller heads. The discovery of precocenes, plant compounds causing precocious metamorphosis [8], enabled “chemical allatectomy”. However, these compounds are blunt instruments [9] that work well in some insects, but are too toxic or too quickly metabolized in others to elicit precocious metamorphosis. Tarrant and Cupp [10] noted that the first-instar larvae of the true bug *Rhodnius prolixus* were “quite refractory” to chemical allatectomy with precocene. Pener and his colleagues treated late embryos or newly hatched first-instar larvae of locusts and cockroaches with precocene and saw precocious appearance of adult features after the second larval molt, but not earlier ([11], [12] and papers cited therein). Yet, the early-larval instar CA secrete JH as shown by transplantation experiments or direct measurements [13]. Significantly, constitutive overexpression of a JH esterase using an actin-Gal4 driver caused precocious metamorphosis in the silkworm [14]. The excess JH esterase specifically inactivates circulating JH. Similar to the loss of CYP15C1 in *mod* mutants, however, overproduction of JH esterase alone was insufficient to force *Bombyx* to enter metamorphosis before they underwent three larval instars [14].

The observations of Daimon et al. are therefore not unprecedented, but their evidence is compelling. The question of the role of JH during early larval instars and the reason for its status quo function at the organismal level becoming apparent only in later instars is now open. A “competence for metamorphosis” requiring some amount of postembryonic growth has been advanced, but this sounds much like a “dormitive virtue”, and needs to be dissected with current molecular tools. Early experiments such as parabiosis and tissue transplantation indicated an immediate competence for metamorphosis (cited by Wigglesworth [15]), a conflict with the results showing delayed competence that was attributed to the persistence of circulating JH. This is not the case in the silkworm, where either loss of JH production [4] or elimination of active JH [14] both result in precocious metamorphosis, but only after it undergoes a minimum of two larval molts. Clearly, factors other than JHs must be at play during the earliest larval instars.

The silkworm appears to be a good model to study this apparent JH-independent growth of the early larval stages, because in this species JH is essential neither during pupal and adult development, nor for reproduction, as evidenced by the viability of the *mod* mutants. Additional loci are known to control the number of larval molts in *Bombyx* [16] and require identification in order to separate JH-dependent from JH-independent effects. Further manipulation of JH synthesis and/or signaling is now possible in *Bombyx*. Inactivation of another enzyme of JH synthesis, the methyl transferase JHAMT [17], should confirm the results obtained with CYP15C1 and prove that the larval JHs cannot be substituted by their non-epoxidized methyl esters. JHAMT RNAi knockdown causes precocious metamorphosis in the beetle *Tribolium castaneum*, clearly establishing this enzyme as essential for the maintenance of larval status [18]. It may also become possible to genetically ablate the CA in the silkworm as was done in *Drosophila* [19], [20]. Manipulation of JH signaling also becomes feasible, as it has recently been shown that insects use a “common core” JH signaling pathway [21] consisting of the bHLH-PAS protein Met [22]–[24] and the transcription factor Kr-h1 [25], [26]. Met emerges as the long-sought JH receptor [27] that controls expression of Kr-h1 to prevent metamorphosis. Responsiveness of Met and Kr-h1 to JH during the earliest larval instars should now be tested. Furthermore, CA cells along with JH appear at or close to dorsal closure in insects, and the role of JH in the transition from embryogenesis to larval stages needs reexamination [28]. With a size propitious for physiological experimentation, a complete genome [29], a thousand races including over 500 mutants, and the tools for targeted gene expression [30] and disruption [31], *Bombyx mori* brings answers to the open questions within reach. The silkworm as a genetic model for insect physiology has truly come of age.
